# The Interplay of Human and *Mycobacterium Tuberculosis* Genomic Variability

**DOI:** 10.3389/fgene.2019.00865

**Published:** 2019-09-18

**Authors:** Wilian Correa-Macedo, Geison Cambri, Erwin Schurr

**Affiliations:** ^1^Program in Infectious Diseases and Immunity in Global Health, Research Institute, McGill University Health Centre, Montreal, QC, Canada; ^2^The McGill International TB Centre, McGill University, Montreal, QC, Canada; ^3^Department of Biochemistry, Faculty of Medicine, McGill University, Montreal, QC, Canada; ^4^Graduate Program in Health Sciences, School of Medicine, Pontifícia Universidade Católica do Paraná, Curitiba, Brazil; ^5^Departments of Human Genetics and Medicine, Faculty of Medicine, McGill University, Montreal, QC, Canada

**Keywords:** tuberculosis, *Mycobacterium tuberculosis* complex group, host–pathogen interaction, genomic variability, gene–genotype interaction, coadaptation

## Abstract

Tuberculosis (TB), caused by the human pathogens *Mycobacterium tuberculosis* (*Mtb*) and *Mycobacterium africanum*, has plagued humanity for millennia and remains the deadliest infectious disease in the modern world. *Mycobacterium tuberculosis* and *M. africanum* can be subdivided phylogenetically into seven lineages exhibiting a low but significant degree of genomic diversity and preferential geographic distributions. Human genetic variability impacts all stages of TB pathogenesis ranging from susceptibility to infection with *Mtb*, progression of infection to disease, and the development of distinct clinical subtypes. The genetic study of severe childhood TB identified strong inborn single-gene errors revealing crucial pathways of vulnerability to TB. However, the identification of major TB-susceptibility genes on the population level has remained elusive. In particular, the replication of findings from candidate and genome-wide association studies across distinct human populations has proven difficult, thus hampering the characterization of reliable host molecular markers of susceptibility. Among the possible confounding factors of genetic association studies is *Mtb* genomic variability, which generally was not taken into account by human genetic studies. In support of this possibility, *Mtb* lineage was found to be a contributing factor to clinical presentation of TB and epidemiological spread of *Mtb* in exposed populations. The confluence of pathogen and human host genetic variability to TB pathogenesis led to the consideration of a possible coadaptation of *Mtb* strains and their human hosts, which should reveal itself in significant interaction effects between *Mtb* strain and TB-susceptibility/resistance alleles. Here, we present some of the most consistent findings of genetic susceptibility factors in human TB and review studies that point to genome-to-genome interaction between humans and *Mtb* lineages. The limited results available so far suggest that analyses considering joint human–Mtb genomic variability may provide improved power for the discovery of pathogenic drivers of the ongoing TB epidemic.

## Introduction

The out-of-Africa migration exposed human populations to changing environments and hence introduced new immunological challenges caused by newly encountered microbial populations. The interaction with these microorganisms, especially human pathogenic species, resulted in strong selective pressures that shaped our immune system (Barreiro and Quintana-Murci, 2010; Nedelec et al., 2016; Quintana-Murci, 2019). Likewise, pathogens experienced selection *via* their need to overcome or avoid host defenses and to be able to spread from host to host or between host and environment. Pathogens and humans have been genetically shaped by this interplay of selective forces. Despite the incomplete mechanistic understanding of how this host–pathogen interplay impacted host and pathogen genetics, new concepts about host–pathogen coevolution have emerged (Brites and Gagneux, 2015).

An illustrative example for the above statements is provided by the adaptation of different strains of *Helicobacter pylori* to human populations of distinct ancestry (Kodaman et al., 2014). The coevolution of *H. pylori*, a main risk factor of human gastric cancers, and its human hosts was studied in two Colombian populations. The two populations displayed different admixture proportions of African, Amerindian, and European ancestries but presented very similar high *H. pylori* colonization levels with strikingly different incidences of gastric cancer. *Helicobacter pylori* isolates affecting both populations were derived from four ancestral lineages originating from Africa, Europe (2×), and Asia. Cancer incidence was found to be lowest when African ancestry of the host was matched with African ancestry of *H. pylori*. In contrast, gastric cancer risk was highest in hosts where the bacterial strain ancestry was divergent from the host ancestry (Kodaman et al., 2014). This work highlighted the importance of considering the coevolution of host and pathogen to better understand clinical progression of diseases involving infectious agents and suggested that resistance and susceptibility to infectious diseases are probably best understood when considering both the genetic make-up of the pathogen and the host. A hotly debated question in the field of tuberculosis (TB) is to what extent a similar coadaptation of *Mycobacterium tuberculosis* (*Mtb*), the major etiological agent of TB, and the human host has occurred. Host-centric genetics studies in TB have provided some insight in host vulnerabilities to *Mtb*. However, the identification of strong genetic drivers of TB susceptibility has remained elusive (Orlova and Schurr, 2017; Abel et al., 2018; Dallmann-Sauer et al., 2018). In this review, we present selected findings from the field of host genetics of TB, describe the results of studies that implicated *Mtb* lineage as susceptibility driver, and focus on results that support a joint effect of both host and *Mtb* genomic variability on TB disease.

## Tuberculosis Disease

Human TB is caused by acid-fast bacilli belonging to the *Mtb* complex (MTBC) group, namely, *Mtb* and *Mycobacterium africanum*. The disease affects mainly the lungs, causing pulmonary TB (PTB), but it can spread to virtually any part of the body leading to distinct clinical phenotypes such as TB meningitis (TBM), a severe form of the disease characterized by progressive meningoencephalitis with necrotizing and granulomatous inflammation leading to obstruction of cerebrospinal fluid passage or infarcts of intracerebral arteries (Thwaites et al., 2013; Zumla et al., 2013). Tuberculosis is an ancestral infectious disease that accounted for an estimated 1 billion deaths in the last 200 years globally (Paulson, 2013), which is broadly consistent with the estimate of 1 billion deaths over the last 2,000 years in Europe only (Kerner et al., 2019). Tuberculosis is generally treatable, yet despite substantial efforts to control the disease, it remains the main cause of deaths by an infectious agent in the world. In 2017, the World Health Organization estimated 10 million new cases resulting in 1.57 million deaths worldwide (World Health Organization, 2018). This is partly due to the emergence of multidrug resistance including rifampicin-resistant *Mtb* for which treatment success remains low. Moreover, the TB control challenge is exacerbated by the HIV epidemic as people living with HIV are at increased risk of becoming infected by *Mtb* and of developing severe clinical forms of TB. In 2017, more than 460,000 new cases of HIV–TB were detected, and approximately 300,000 deaths occurred among HIV-positive patients due to TB (World Health Organization, 2018).

## Human Genetics of TB

Several biological factors contribute to susceptibility to TB. Among these, host genetics plays a clear role in modulating risk for development of TB and its clinical forms. Early evidence for the impact of host genetics on TB susceptibility came from the observation of familial aggregation (Puffer, 1944) and a higher concordance rate of TB in monozygotic compared to dizygotic twins (Kallmann and Reisner, 1943). Likewise, an often-quoted example for the role of host genetics in TB risk is the Lübeck accident, which occurred in 1929 (Fox et al., 2016). Due to the accidental contamination of bacillus Calmette–Guérin (BCG) vaccines, 251 newborns received *Mtb*-contaminated batches of BCG. Of the total number of vaccinated babies, 228 (90.8%) developed diverse forms of clinical TB. A year after the accident, 77 infants had died, and 69 of these deaths were confirmed to be related to TB. Surprisingly, 23 infants (9.2%) did not present any clinical signs for the disease, and 68% of the clinically confirmed TB cases progressed to spontaneous cure. These events suggested that host genetic factors played a role in TB susceptibility and severity of the disease. Interestingly, it was also shown that high levels of contamination overrode intrinsic TB resistance factors (Fox et al., 2016). These older studies along with more recent examples provide the persistent motivation for the investigation of host genetics in TB (Abel et al., 2018).

A widely accepted model for TB pathogenesis assumes two main stages of the disease process. In step 1, exposed persons become infected with *Mtb* without any clinical signs of disease. These individuals are distinguished by converting to positivity in immune assays such as the *in vivo* tuberculin skin test (TST) or *ex vivo* release of interferon γ by blood cells in response to *Mtb* antigen (IGRA tests). Such persons are thought to carry *Mtb* bacilli and to suffer from latent TB infection (LTBI). In step 2, a subset of those who are classified as LTBI will develop TB, mostly within the first 2 years of LTBI conversion (Behr et al., 2018). In TB, natural resisters to infection have been inferred from heavy *Mtb* exposure settings in which subjects failed to convert to LTBI, and this infection resistance phenotype has been shown to have a genetic component (Orlova and Schurr, 2017; Abel et al., 2018; Simmons et al., 2018; Dallmann-Sauer et al., 2018). Studies of resistance to TST conversion have consistently implicated chromosome region 11p14, both in families from a hyperendemic area in South Africa (logarithm of the odds [LOD] score = 3.81, *P* = 1.4 × 10^−5^) (Cobat et al., 2009) and in a sample of ethnically mixed French families with low TB endemicity (LOD score = 3.65, *P* = 2.08 × 10^−5^; combined sample populations: LOD score = 4.54, *P* = 2.4 × 10^−6^) (Cobat et al., 2015). In an Ugandan population, chromosome regions 2q21-2q24 and 5p13-5q22 reached suggestive *P* values (*P* = 3 × 10^−4^ and 5 × 10^−4^, respectively) for linkage with persistent TST negativity (Stein et al., 2008), and in a Ghanaian sample, *IL10* haplotypes were associated with low circulating cytokine levels, which were enriched in TST-positive versus TST-negative persons (odds ratio [OR] = 2.09, *P* = 1.7 × 10^−2^) (Thye et al., 2009).

In PTB, chromosome region 8q12–q13 was linked to disease in 96 Moroccan families (LOD score = 3.49, *P* = 3 × 10^−5^) (Baghdadi et al., 2006), and subsequent fine mapping revealed association between PTB and a cluster of single-nucleotide variants (SNVs) in high linkage disequilibrium overlapping the *TOX* gene. This SNV cluster initially failed to be significantly associated with PTB in a Malagasy sample. However, stratifying the analysis for patients with age at onset of TB younger than 25 years replicated the association for rs2726600 in *TOX* (Grant et al., 2013). Moreover, a PTB-susceptibility locus has been detected on chromosome region 20q13 by a genome-wide linkage study in Malawi and South African families (LOD score = 3.1, *P* = 8 × 10^−5^) (Cooke et al., 2008). This linked region was further screened with 40 SNVs, and association tests using selected markers in combined samples found protective associations for *MC3R* (OR = 0.67, *P* = 4 × 10^−3^) and *CTSZ* (OR = 0.49, *P* = 9 × 10^−3^) with PTB. Additional genome-wide linkage studies reached suggestive significance on chromosome 20q13 for families from Uganda (Stein et al., 2008) and Thailand (Mahasirimongkol et al., 2009). An independent study employing a South African population found association of variants present in *MC3R* and *CTSZ* with PTB (*P* = 4 × 10^−4^ and <1 × 10^−4^, respectively) (Adams et al., 2011), but in an Iranian sample, only *MC3R* variants were associated (OR = 1.56, *P* = 5 × 10^−3^) (Hashemi et al., 2013). Chromosome 11 also harbors evidence of genetic factors controlling PTB. A genome-wide association study (GWAS) of a Ghanaian sample found the variant rs2057178 located on region 11p13 downstream of the *WT1* gene associated with protection from TB (OR = 0.77, *P* = 2.63 × 10^−9^). This association was replicated with diminished effect size in Gambian (OR = 0.8, *P* = 4.87 × 10^−4^) and Russian samples (OR = 0.91, *P* = 0.02) (Thye et al., 2012). Two independent GWAS replicated marker rs2057178 in two samples from South Africa (OR = 0.62, *P* = 2.71 × 10^−6^) (Chimusa et al., 2014) and Morocco (OR = 0.78, *P* = 0.043) (Grant et al., 2016).

To date, the HuGE navigator’s phenopedia search engine lists more than 200 genes with variants that had been reported by candidate gene studies or GWAS as being associated with either susceptibility or protection to PTB. The top genes in the number of publications reporting significant genotypic associations are *HLA-DRB1*, *VDR*, and *SLC11A*. Variants in these genes were almost exclusively detected by candidate gene approach (Stein et al., 2017). *HLA-DRB1* is the exception given that it has been recently identified in a GWAS employing a large Icelandic population of 277,643 controls and 8,162 cases (Sveinbjornsson et al., 2016). A shared aspect of genetic studies of TB susceptibility is the poor replication of risk variants across independent studies and different populations. Associations of variants in the *SLC11A1* gene are among the most stable findings across different populations, although with varying effect sizes (Stein et al., 2017; Abel et al., 2018).

One of the strongest protective genetic effects for PTB detected at the population level has been described for chromosome region 5q33.3 in a combined sample of Ugandan and Tanzanian HIV patients (Sobota et al., 2016). After following up HIV patients from TB hyperendemic areas, 314 HIV-infected patients remained free of TB, whereas 267 developed active disease. Contrasting these two groups in a case-control GWAS identified the risk SNV rs4921437 located in an intron of the *UBLCP1* gene at 51 kb downstream from *IL12B* (OR = 0.37, *P* = 2.11 × 10^−8^). The variant mapped to an annotated histone modification site, suggesting an impact on RNA expression levels and potentially an important role of epigenetic effects in TB susceptibility. However, no functional data for this SNV with either gene were reported (Sobota et al., 2016). The implication of *IL12B* alleles as genetic vulnerability to TB is interesting due to its link to Mendelian susceptibility to mycobacterial disease (MSMD). Patients with MSMD, who are characterized by severe clinical disease caused by weakly virulent mycobacteria, often harbor deleterious mutation in genes controlling *IFNG*/*IL12*-mediated immunity that can also predispose to TB (Abel et al., 2018). While individual mutations in MSMD genes are rare, it has been shown that a collection of loss of function mutations in MSMD genes can explain a substantial proportion of TB cases (Alcais et al., 2005). Another example for an important role of loss of function mutations in genes of the IFNG/IL12/IL23 circuitry is provided by *TYK2* deficiency. Homozygosity for the common *TYK2* P1104A allele is a strong risk factor for TB explaining an estimated 1% of TB deaths in Europe (Boisson-Dupuis et al., 2018; Kerner et al., 2019). Interestingly, the data suggested a strong selective pressure against the susceptibility allele since the frequency of P1104A in Europeans has decreased from 9% to 4.2% over the last 4,000 years (Boisson-Dupuis et al., 2018). Given its very strong impact on TB risk, the P1104A allele does provide a good opportunity to test for an interaction with *Mtb* strains and possible coadaptation.

An unexpected outcome of genetic studies trying to pinpoint TB-susceptibility genes was the limited success in the identification of common (minor allele frequency >1%) alleles as genetic risk factors for TB susceptibility. It is possible that genetic heterogeneity resulting from the combination of different common and rare DNA variants may give rise to a complex model of inheritance in which candidate gene or GWAS approaches fail to capture major genetic susceptibility drivers (Abel et al., 2018; Dallmann-Sauer et al., 2018). In this respect, it remains to be seen whether the application of whole genome or exome sequencing including the approach of focusing on extreme phenotypes and less common variants with strong effect, such as described for *TYK2*, can provide further insights. Perhaps, an equally important issue is phenotype definition. For example, the clinical definition of PTB may encompass distinct disease stages that are under distinct genetic control as has been shown in a mouse model of mycobacterial infection (Di Pietrantonio et al., 2010). Aside from host-centric characteristics, the genetic variability of the *Mtb* strain infecting subsets of subjects within a sample may partially account for the low success of genetic studies, from the low effect findings to poor marker consistency between different populations. Mounting experimental evidence suggests that MTBC lineages interact differently with the immune system potentially leading to distinct clinical manifestations (Tientcheu et al., 2017), and some genetic studies have been proof of concept that considering *Mtb* lineage in the analysis allows the finding of signals otherwise missed (Caws et al., 2008).

## *Mtb* Lineage Characteristics and Geolocalization

The MTBC group, of which *Mtb* is part, comprises human- and animal-adapted species capable of causing disease in a wide range of hosts. The MTBC group consists of bacterial species with high DNA sequence similarity; however, genomic markers exist that allow classification of mycobacterial lineages (Brites and Gagneux, 2017). Within human-adapted strains, *M. tuberculosis sensu stricto* and *M. africanum* are divided in seven phylogenetic lineages, which cluster into preferred geographical locations. *Mycobacterium tuberculosis* strains are divided into lineages 1 to 4, along with lineage 7, while *M. africanum* strains fall into lineages 5 and 6. In terms of geographical spread, lineages 5 to 7 are more restricted occurring mainly in West Africa (lineages 5 and 6) and Ethiopia (lineage 7). The distribution of lineages 1 and 3 is more diffuse, with lineage 1 mainly found in proximity to the Indian Ocean (hence also known as Indo-Oceanic strain), whereas lineage 3 is more commonly found in East Africa as well as Central and South Asia. The remaining lineage 2 (alias East Asia lineage) and lineage 4 (alias Euro-American lineage) are widely distributed globally. The Beijing family of *Mtb* strains are prominent members of lineage 2, whereas Harlem strains belong to lineage 4. In addition, strains of lineages 2, 3, and 4 carry the genomic deletion TBD1 and are referred to as modern strains (Brites and Gagneux, 2017).

## Evidence for *Mtb* Lineage Association With Human Ethnicity

The geographical distribution of lineages leads to a clear association with specific human populations (Gagneux et al., 2006). This sympatric pathogen–host relationship is reflective of comigration and coexpansion of human populations and *Mtb* (Comas et al., 2013; Luo et al., 2015; Stucki et al., 2016). The concept of *Mtb* and human host coadaptation gained additional support by a study of two Swiss cohorts. Subjects were followed for 8 and 10 years, and preferential infection by lineage 4 (Euro-American) was found in individuals born in Europe. Conversely, non-Europeans were more likely to be infected by other strains. The preferential host ethnicity–*Mtb* lineage relationship was maintained despite social mixing of subjects and after correction by age and sex (Fenner et al., 2013). These observations were consistent with other studies that also reported preferential associations of bacterial lineage with the phylogeographic region where TB patients were born even in areas with high pathogen and host ethnicity admixture (Hirsh et al., 2004; Reed et al., 2009; Pareek et al., 2013; Rasigade et al., 2017; Guthrie et al., 2018). For *M. africanum*, coadaptation with specific African subpopulations has been hypothesized as cause for its much restricted geographical distribution (Asante-Poku et al., 2015; Otchere et al., 2018); however, additional molecular epidemiology studies are needed. A major challenge in the interpretation of these results is the possibility of social contact bias, in which subjects from the same ethnicities tend to interact more frequently, hence increasing the chances for preferential ethnicity-lineage pairings. However, the case for true association is strengthened by the observation that preferential pairing of host ethnicity and *Mtb* lineage is not maintained in the case of HIV–TB coinfection, which occurs in the context of low CD4 T-cell counts (Fenner et al., 2013). Likewise, alterations in the transmission dynamics in defined geographical areas by introduction of foreign, more virulent strains can disrupt preferential pairings. For example, Beijing family strains (lineage 2) were shown to be overtransmitted in a Kinh Vietnamese population in contrast to lineage 1. The increased virulence of Beijing strains in this setting was supported by shorter times of progression to active disease, wider geographical dispersal, and higher incidence of infection in younger individuals (Holt et al., 2018).

## The Interplay of *Mtb* Lineage and the Human Host

It is known from murine studies that infection with different strains of Mtb results in significant differences in survival times for a given mouse strain. For instance, immunocompetent CB-17 mouse strains infected with Mtb HN878 (Lineage 2) resulted in increased mortality when contrasted against NHN5, HN60, CDC1551 and H37Rv strains (Manca et al., 2001). Likewise, when infecting different strains of inbred mice with the same Mtb strain, there are significant differences in survival times (Schurr and Kramnik, 2008). Variable disease outcomes promoted by *Mtb* lineages have also been demonstrated with common marmosets, an animal model that more closely resembles human TB. In contrast to mouse models, these animals develop lung pathology and infection with ancient (lineage 6) or modern lineages (2 and 4), resulting in different profiles of pulmonary damage, bacterial dissemination, and survival (Via et al., 2013). Distinct patterns of host responsiveness have also been observed for human studies. For example, a recent study assessed bacterial virulence of *Mtb* obtained from 153 Vietnamese TB patients employing a monocyte-derived macrophage lysis model. A high degree of macrophage lysis (i.e., high virulence) was associated with higher bacterial concentrations in sputum. High virulence strains also replicated faster in the macrophages and induced lower secretion of tumor necrosis factor α (TNF-α) and IL-6, but higher production of IL-1β. This high virulence bacterial phenotype was strongly associated with lineage 2 strains (Tram et al., 2018). However, this study did not address a possible “fit” between host and *Mtb* genetic background. The latter question was addressed by a human study, which analyzed cytokine production by monocyte-derived macrophages obtained from members of three ethnicities following infection with different strains of *Mtb*. In univariate analyses, both Filipino ethnicity and *Mtb* strain CDC1551 were significant predictors of cytokine production. However, there was no evidence for significant interaction between ethnicity and *Mtb* strain possibly arguing against coadaptation in this sample (Nahid et al., 2018). Considering that a large number of tests were conducted (nine cytokines vs. four *Mtb* strains) in three groups with only 45 subjects per group, it is clear that power to detect such interactions was modest. Similar studies employing larger sample size and live bacterium challenges may provide different results.

A study of TB patients of Eurasian versus African ancestry (83 vs. 43 individuals) from London, UK, took a different approach. A large number of hematological parameters and soluble factors were determined at time of initiation of TB treatment, and five were found to be significantly different among the two ethnic groups. Similarly, whole blood was stimulated with *Mtb* antigens, soluble factors were determined, and IL-1RA and IL-12 concentrations were found significantly different between the two ethnicity groups (Coussens et al., 2013). However, in agreement with earlier studies, it was shown that these ethnicity-specific differences in immune responsiveness were independent of the lineage of the causative *Mtb* strain arguing against an interaction of strain and host genomes. In contrast, the observation of a strong genotypic impact of a VDBP polymorphisms on the response profile further supported the hypothesis that differences in immune responsiveness are primarily driven by host genetic variability (Coussens et al., 2013). While there was no significant impact of *Mtb* lineage by univariate analysis, it is possible that a significant interaction effect might have been detected; however, this was not tested. The genetic control for differences in immune response between European or African ancestry was investigated by genome-wide approaches, and extensive differences in inflammatory responses were detected by RNA sequencing (Nedelec et al., 2016). Along the line of unbiased genome-wide approaches, a detailed human–Mtb protein–protein interaction map has recently been described (Penn et al., 2018). Unfortunately, *Mtb* lineage- or strain-specific data for the map are not yet available.

A common observation among studies was the reduced/delayed triggering of a proinflammatory response upon infection of host cells with “modern lineages” (lineages 2–4) and a corresponding increased intracellular growth rate by lineages 2 and 4 *Mtb* strains (reviewed in (Coscolla and Gagneux, 2014)). However, strong heterogeneity was observed across studies. For example, Krishan et al. demonstrated a higher production of proinflammatory cytokines (i.e., TNF and IL1β) in macrophages elicited by East Asian/Beijing and Indo-Oceanic strains in comparison to Euro-American lineages, whereas this modulation was less pronounced in dendritic cells (Krishnan et al., 2011) and absent from peripheral blood mononuclear cells (Portevin et al., 2011). By contrast, another study described low and high cytokine induction by lineages 1 and 2 in contrast to lineage 4, respectively (Reiling et al., 2013).

Differences in transmissibility of *Mtb* strains from patients to contacts have been noted repeatedly (Malik and Godfrey-Faussett, 2005). For example, lineages 2 and 4 are often referred to as the most transmissible strains, which could reflect the modulation of host immune response and hence increased virulence (Parwati et al., 2010). Indeed, a meta-analysis identified lineage 2 as the only lineage with a significantly increased risk of transmission employing lineage 4 strains as reference group (Wiens et al., 2018). However, there was substantial heterogeneity of results across studies. For example, lineage 2 was not overtransmitted in geographically diverse settings in Vietnam, The Gambia, Canada (Montreal and Alberta), and South Africa (Cape Town) (de Jong et al., 2008; Marais et al., 2009; Albanna et al., 2011; Buu et al., 2012; Langlois-Klassen et al., 2013). By contrast, lineage 3 (East African Indian strains) was undertransmitted in a Canadian study, even when analysis was conducted in patients where the strains are most frequently encountered (Albanna et al., 2011). This is in agreement with the results of the meta-analysis, which found lineage 3 strains presented reduced risk of transmission in Europe and Americas (Wiens et al., 2018). The same meta-analysis also found substantially increased risk of overtransmission of lineage 2 strains in East Asia (compared to other regions), whereas lineage 1 strains were associated with overtransmission only in East Asia (Wiens et al., 2018). It is not known if geographical differences in risk of transmission of certain *Mtb* strains reflect coadaptation of *Mtb* to specific human populations, other epidemiological or demographic factors, or the emergence of lineage substrains with greatly increased virulence and transmission potential (Rajwani et al., 2017).

Given that extrapulmonary TB cases present a lower risk for transmission of TB, we might expect preponderance of PTB as a result of coadaptation. Indeed, transmission potential has been linked to different pathways of pathogenesis (Verma et al., 2019). A study from Birmingham and Solihull, UK, reported increased extrathoracic TB for the East Asian lineage (lineage 2), but not for East African Indian lineage (lineage 1), using strains of the Euro-American lineage (lineage 4) as reference (Pareek et al., 2013). Similarly, Beijing strains (lineage 2) resulted in higher frequency of extrapulmonary TB compared to non-Beijing strains (Kong et al., 2007). By contrast, a retrospective study (2004-2015) in British Columbia, Canada, found that strains of the Indo-Oceanic lineage (lineage 1) were significantly enriched among cases with extrapulmonary TB compared with all other lineages (Guthrie et al., 2018). Consistent with the Canadian observations, a large US study of 32,000 TB cases with known *Mtb* lineage and disease site found that relative to all other lineages, lineage 2 (East Asian) was underrepresented among extrapulmonary TB cases. These results remained unchanged when adjusted for ethnicity or place of birth of the index case and collectively argued against a genetic interaction of *Mtb* and the human host in the determination of the clinical site of TB. However, given the conflicting results from different studies, it seems possible that lineage and ethnicity are too crude classifications and that a higher-resolution approach may be required.

Overall, these findings show a major impact of *Mtb* lineage on TB pathogenesis, which is a necessary factor for the coevolution of *Mtb* and its human host. Curiously, the strongest data in favor of human–Mtb coevolution arose in a host-free study. In a landmark study, Comas et al. (2010) found direct evidence for the action of selective pressures on the Mtb genome. Intriguingly, it was found that the strongest signs of selection were favoring conservation of T-cell epitopes across a large panel of Mtb strains (Comas et al. 2010). The most parsimonious explanation for this finding was that Mtb has adapted to human T-cell immunity and that aspects of the host T-cell response are beneficial for the transmission of Mtb (Comas et al., 2010; Sassetti and Rubin, 2010). However, much of the *Mtb* lineage impact on TB pathogenesis appears to transcend host genetics. For example, lineage 2 increases TB transmission and together with lineage 4 is more likely to cause severe pulmonary infection, irrespective of age, sex, and ethnicity (Rasigade et al., 2017; Wiens et al., 2018).

## Evidence for Host Gene/*Mtb* Genotype Interaction

To address the problem of strain-specific genetic control in susceptibility to mycobacterial infection, a study employed recombinant congenic mice infected with BCG Russia and BCG Pasteur. In this design controlled for infection dosage and host genetic makeup, a strong linkage hit on chromosome 1 was identified for spleen-specific bacterial burden for both BCG Pasteur and BCG Russia infection. Interestingly, additional BCG Russia–specific linkage hits were detected on chromosomes 11 and 13 (Di Pietrantonio et al., 2010). By analyzing the host pathogen contribution to widely used read-outs of host responsiveness to mycobacteria such as secretion of interferon γ, IL-4, IL-6, and IL-12, it was possible to detect significant interaction of host genetics and pathogen strain on cytokine production (Di Pietrantonio et al., 2011). These results established that under strictly controlled experimental conditions strain-specific differences in host susceptibility can be detected and that specificity exists in the host–mycobacteria interaction (Di Pietrantonio and Schurr, 2013).

In human populations, careful studies were able to demonstrate evidence for a human–Mtb genetic interplay, despite the experimental challenges such as disease heterogeneity among cases or low study power as consequence of sample size. These results are particularly encouraging, considering the intrinsic challenges of transmission studies and the complexity of TB pathogenesis. The first report for lineage-dependent SNV association with human TB was based on a case-control Vietnamese sample with mixed cases of PTB and TBM. In this work, contrasting controls to a subset of patients infected with the East Asian/Beijing lineage revealed the SNV T597C in *TLR2* as a risk factor for TB. Further study of the lineage-dependent effect showed that the association was driven by the smaller subset of TBM patients infected with the Beijing genotype (OR = 1.91, *P* = 1 × 10^−3^) (Caws et al., 2008). Expanding the previous studied sample and applying a discovery and validation case-control design, two *TLR9* SNVs in strong LD were found associated with a combined TB phenotype, with rs352142 displaying the most significant association signal (OR = 2.33, *P* = 4 × 10^−3^). To test for a lineage-specific effect, discovery and validation samples were combined, and rs352142 showed a trend of association with a subsample infected by *Mtb* belonging to the Indo-Oceanic or Euro-American lineages (χ^2^ = 3.67, *P* = 0.056) (Graustein et al., 2015). In Vietnamese subjects, SNV markers in the macrophage receptor *MARCO* were found associated with increased risk to PTB (rs2278589, OR = 1.6, *P* = 1 × 10^−3^; rs6751745, OR = 1.4, *P* = 9 × 10^−3^). Interestingly, a significant association was found between these two markers and the Beijing lineage, which was strongest under a heterozygous model (OR = 1.7, *P* = 1 × 10^−3^; and OR = 1.5, *P* = 1 × 10^−2^, respectively) (Thuong et al., 2016). Testing of host genotypes with preferential association of *Mtb* lineage was conducted in two additional studies. An Indonesian study identified a strong association of *SLC11A1* alleles with Beijing strains genotype, whereas a study of South Africans identified HLA class I alleles that were associated with strains of the Beijing group. While associations of host genotypes with *Mtb* lineage support the concept of host *Mtb* coadaptation, the reported results should be considered tentative until a better understanding of the implicated alleles with TB risk has been established (van Crevel et al., 2009; Salie et al., 2014).

In a Ghanaian sample, testing association of 18 polymorphisms in the *IRGM* gene with PTB resulted in no significant findings for the total sample. However, stratifying patients by *Mtb* or *M. africanum/Mycobacterium bovis* strain detected a protective association of rs9637876 (5′ UTR in *IRGM*) against *Mtb* (OR = 0.66, *P* = 4.5 × 10^−3^). Further study revealed the protective effect of the *IRGM* variant to be significantly stronger for *Mtb* strains belonging to the Euro-American lineage (Intemann et al., 2009). Reinvestigating the Ghanaian case-control cohort revealed rs1800451 in *MBL2* to be associated with protection from PTB caused by *M. africanum* but not by *Mtb* (OR = 0.6, *P* = 0.008) (Thye et al., 2011). Following the findings for *IRGM* in West Africa, a study conducted in Indonesians failed to associate SNVs in *IRGM* and 13 additional autophagy-related genes with TB. Analysis stratified by patient *Mtb* isolate lineage reached borderline nominal significance for two genes, but remained non-significant after multiple-testing correction (Songane et al., 2012). To date, the only GWAS that took lineage into account was conducted in a combined set of two Thai population samples. Total sample screening failed to find SNVs associated with TB. However, stratified analysis based on *Mtb* lineage found association of rs1418425 (OR = 1.62, *P* = 1.58 × 10^−7^, chromosome region 1p13, intergenic region of *CD53* and *LRIF1*) in patients infected with non-Beijing lineage strains (61% of patients, 49% being infected by East African Indian/Indo-Oceanic lineage). Further stratification based on non–Beijing-infected subjects older than 45 years yielded more significant results (OR = 1.74, *P* = 2.54 × 10^−8^) (Omae et al., 2017). Like the *Mtb* lineage-dependent host genotype associations, these results need to be replicated in an independent sample to confirm the validity of the stratification approach.

## Conclusion

While intuitively attractive and supported by conceptually consistent observations, strong data showing *Mtb*-human coevolution are still missing. There are data that show how infectious pathogens have shaped the human genome with major impact on genes encoding proteins of the innate immune system. Yet, the identification of the specific pathogens that underlie selection of specific human variants has proven more difficult. Well-powered studies specifically designed to address the role of human–Mtb coadaptation are needed to follow up on the general concept, and such studies need to be complemented with specific examples of gene–gene interaction between the human host and *Mtb* isolates. From the human side, it may be interesting to focus on studies employing strong dominant acting *Mtb* resistance alleles, most likely to be found by studying *Mtb* infection, or strong recessive acting susceptibility alleles, such as provided by the *TYK2* example. On the bacterial side, a follow-up on protective epitopes stratified by human hosts may be equally informative. In general terms, it seems likely that the stratification by bacterial linages is too crude to allow significant conclusions. Future studies likely will need to consider high-resolution genomic substrains (Coll et al., 2014; Stucki et al., 2016), which, unfortunately, will increase the required sample size. While the challenges are daunting, they may well be worth the effort. The study of biological problems and their relevance for human health has provided numerous examples that ignoring the powerful forces of evolution may not be a wise choice.

## Author Contributions

WC-M and GC contributed equally to the literature review and manuscript writing. ES contributed to the writing of the manuscript. All authors read and approved the final manuscript.

## Funding

Research in the authors’ laboratory is supported by a foundation grant from the Canadian Institute of Health Research (grant 143332).

## Conflict of Interest Statement

The authors declare that the research was conducted in the absence of any commercial or financial relationships that could be construed as a potential conflict of interest.
